# Expression of HCMV-Encoded miRNA in Subjects Acutely Coinfected with HIV: Correlation with Inflammation and Immune Activation

**DOI:** 10.3390/ijms26125673

**Published:** 2025-06-13

**Authors:** Elisabetta Lazzari, Gabriella Rozera, Rozenn Esvan, Roberta Gagliardini, Valentina Mazzotta, Annalisa Mondi, Luigi Federici, Enrico Girardi, Andrea Antinori, Fabrizio Maggi, Isabella Abbate

**Affiliations:** 1 Laboratory of Virology, National Institute for Infectious Diseases Lazzaro Spallanzani-IRCCS, 00149 Rome, Italy; elisabetta.lazzari@inmi.it (E.L.); luigi.federici@inmi.it (L.F.); fabrizio.maggi@inmi.it (F.M.); isabella.abbate@inmi.it (I.A.); 2 Clinical Department, National Institute for Infectious Diseases Lazzaro Spallanzani-IRCCS, 00149 Rome, Italy; rozenn.esvan@inmi.it (R.E.); roberta.gagliardini@inmi.it (R.G.); valentina.mazzotta@inmi.it (V.M.); annalisa.mondi@inmi.it (A.M.); andrea.antinori@inmi.it (A.A.); 3 Scientific Direction, National Institute for Infectious Diseases Lazzaro Spallanzani-IRCCS, 00149 Rome, Italy; enrico.girardi@inmi.it

**Keywords:** miRNA, HCMV, HIV, inflammation, VEGF

## Abstract

Human cytomegalovirus (HCMV) coinfection is associated with a faster HIV disease progression and adverse clinical outcomes. HCMV-encoded miRNA expression, in individuals acutely infected with HIV (AHI), compared to those with HCMV monoinfection, was investigated in relation to viral replication and inflammation/immune activation. Sixteen individuals with AHI coinfected with HCMV were analyzed at serodiagnosis (T0) and after 6 (T1) and 12 (T2) months of antiretroviral therapy initiated within one week from serodiagnosis. Fourteen HCMV monoinfected subjects were also studied. Plasma RNA was reverse-transcribed and amplified with a panel designed to detect 14 different HCMV-microRNAs (miRNAs). VEGF-A and IP-10 plasma levels were quantified using ELISA. Except for hcmv-miR-70-3p, detected in all subjects, hcmv-miR-UL112-3p, hcmv-miR-US25-1-5p, hcmv-miR-US25-2-3p, hcmv-miR-US4-5p, hcmv-miR-US5-1, hcmv-miR-US5-2-3p, hcmv-miR-UL36-3p, and hcmv-miR-UL36-5p were significantly more frequently detected when HCMV DNA was present (lytic infection). In latent HCMV infection, hcmv-miR-UL22A-5p and hcmv-miR-UL148D were more frequently observed in HIV/HCMV-coinfected individuals, compared to mono-HCMV infection. Hcmv-miR-UL22A-5p and hcmv-miR-US33-5p showed a direct correlation with HIV-1 RNA. Notable positive correlations between hcmv-miR-UL22A-5p and the interferon-gamma-inducible protein 10 (IP-10), as well as between hcmv-miR-UL148D and the vascular endothelial growth factor A (VEGF-A), were also observed. HCMV-miRNA expression varies between lytic and latent infection and differs in HIV coinfection. In HCMV/HIV coinfection, increased levels of hcmv-miR-UL148D, associated with VEGF-A production, seem to be less linked to HIV viremia with respect to hcmv-miR-UL22A-5p and hcmv-miR-US33-5p. A deeper understanding of HCMV-encoded miRNA biology may facilitate the comprehension of HCMV/HIV coinfection pathogenetic mechanisms.

## 1. Introduction

According to the microRNA (miRNA) database (www.mirbase.org, last consulted on 14 March 2025), 26 viral miRNAs encoded by the human cytomegalovirus (HCMV) have been identified. These microRNAs are generated by 15 different precursors, and their target molecules have been determined [1,2]. MiRNAs encoded by HCMV are dispersed throughout the HCMV genome and play a significant role in modulating both viral transcription and host transcription, primarily via gene silencing mechanisms. Numerous studies have indicated that HCMV-encoded miRNAs may exhibit differential expression during various phases of HCMV infection: certain miRNAs appear to be linked to active viral replication, whereas others are implicated in the establishment of viral latency within the host [3,4]. The substantial expression of miRNAs among HCMV transcripts implies their considerable biological importance during infection. Evidence suggests that HCMV-encoded miRNAs may contribute to the pathogenesis of human diseases. Consequently, due to their functional significance, specific viral miRNAs, along with numerous human miRNAs, have been recognized as potential biomarkers applicable across different medical fields [5]. Coinfection with HCMV is known to accelerate the progression of HIV disease in affected individuals. In people living with HIV (PLWH), HCMV coinfection is associated with a heightened risk of adverse clinical events, with cardiovascular outcomes being the most frequent [6]. HIV infection correlates with increased levels of systemic inflammatory mediators, such as IL-6 and interferon-gamma-inducible protein 10 (IP-10), also known as C-X-C motif chemokine ligand 10 (CXCL10), along with chronic immune activation, which is evidenced by the persistent activation of platelets, monocytes, and T cells [7]. The relative contribution of these effects from HIV, HCMV, or their potential synergistic interaction in HIV/HCMV coinfection remains uncertain. HCMV presence is frequently linked to vascular damage [8], with significant impacts on endothelial cells arising both from direct cytopathic damage due to viral replication and indirect immune-mediated mechanisms. This study analyzed HCMV-encoded miRNA expression in HIV subjects acutely infected (AHI), compared to those only infected with HCMV. It also evaluated correlations between HCMV miRNA expression, viral replication, and inflammation markers before and after one year of early antiretroviral treatment (ART). Specifically, IP10 and vascular endothelium growth factor (VEGF) were examined due to their role in systemic inflammation and impact on endothelial cells [9], a primary target of HCMV-related pathogenesis in HCMV/HIV coinfection [6,10].

## 2. Results

### 2.1. HCMV-Encoded miRNA Expression in Lytic and Latent HCMV Infection

The demographics and virological features of HCMV/HIV-coinfected and HCMV-monoinfected subjects, with their respective medians and interquartile ranges (IQRs), are described in Table 1A,B.

All subjects tested negative for HCV and HBV markers, except for one subject in the HCMV-monoinfected group, who showed positive antibodies against the HBV core antigen. Individuals with AHI had a median (IQR) of 420 (264–545) CD4 T cell counts and 6.65 (5.13–7.12) Log HIV-1 RNA copies/mL. At T1, half of the subjects showed an HIV-1 RNA > 30 copies/mL, whereas only 1 out of 12 subjects showed this by T2. During the first 6 months of follow-up (T0 vs. T1), among subjects with quantifiable viremia, a rapid and significant reduction in HIV-1 RNA was observed (*p* = 0.008). In the HCMV-monoinfected group, 10 non-viremic individuals were healthy donors, while 4 were HCMV viremic. Among these, two subjects were solid organ transplanted patients with HCMV reactivation (one of them as mentioned above was HBV-positive), one was a woman with an HCMV primary infection, and the last one was a man with HCMV reactivation in the course of a bacterial infection.

The detection of HCMV DNA in plasma, indicative of the production of new viral particles, was utilized as a marker for lytic HCMV infection. Latent infection was inferred based solely on the presence of anti-HCMV-specific IgG. At baseline, HCMV DNA was identified in 2 out of the 16 AHI participants (<2.2 and 5.5 Log IU/mL, respectively). During follow-up, transient low-level HCMV reactivations were observed in two different subjects: one exhibited low-level HCMV reactivation at both T1 and T2 (HCMV DNA detected <293 IU/mL at both times), while the other displayed HCMV DNA levels of 432 IU/mL at T1 only. In the HCMV-monoinfected control group, 4 out of 14 individuals exhibited HCMV DNA viremia, with a median (IQR) of 3.77 (3.39–5.16) Log HCMV DNA IU/mL.

**Figure 1:**
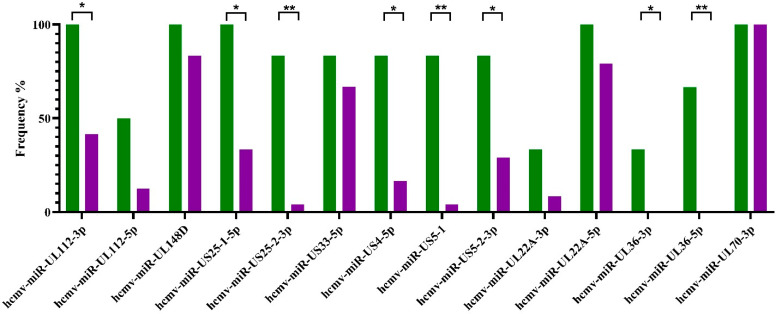
HCMV miRNA detection rates in lytic and latent infections. The y axis displays the observed frequencies, and the x axis shows the fourteen HCMV-encoded miRNAs analyzed. Lytic infections are displayed in green, while latent infection is shown in purple. Significant differences in the miRNA frequencies between the two groups are reported with * *p* < 0.05 and ** *p* < 0.001.

The first analysis aimed to identify distinctive patterns of HCMV-encoded miRNAs in lytic vs. latent HCMV infection among all subjects, combining both HCMV-monoinfected and HCMV/HIV-coinfected individuals (Figure 1). The results showed that hcmv-miRUL112-3p, hcmv-miR-US25-1-5p, hcmv-miR-US25-2-3p, hcmv-miR-US4-5p, hcmv-miR-US5-1, hcmv-miR-US5-2-3p, hcmv-miR-UL36-3p, and hcmv-miR-UL36-5p were more frequently detected in lytic infections, with no statistical difference between mono- and coinfected subjects (*p* > 0.05 for all HCMV-encoded miRNAs). Hcmv-miR-70-3p was detected in all subjects.

### 2.2. Expression of HCMV-Encoded miRNA During Latent Infection in Subjects Monoinfected with HCMV and Coinfected with HCMV/HIV

**Figure 2:**
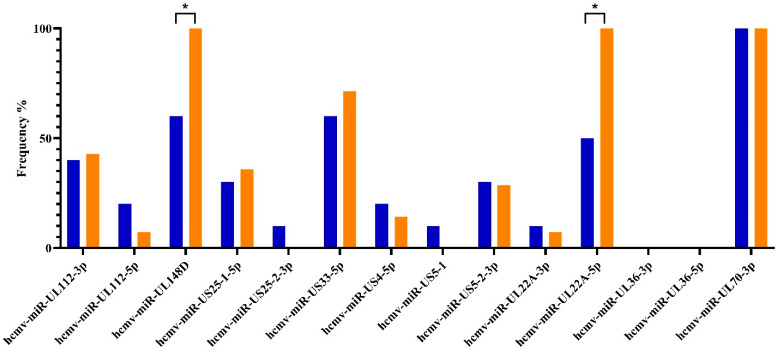
HCMV miRNA detection rates in subjects with HCMV only and those with HIV coinfection. The y axis displays the percentages of the observed *frequencies*; the x axis shows the fourteen HCMV-encoded miRNAs analyzed. HCMV monoinfections are represented in blue, while HCMV/HIV coinfections are in orange. Significant differences in the miRNA frequencies between the two groups are reported with * *p* < 0.05.

During the latent HCMV phase, hcmv-miR-UL22A-5p and hcmv-miR-UL148D were more common in HIV/HCMV-coinfected subjects than in those with only HCMV (Figure 2). The higher frequencies of hcmv-miR-UL148D in the AHI group were consistent at all follow-up times (100% detection at both T1 and T2, *p* = 0.019 and *p* = 0.035). For hcmv-miR-UL22A-5p, the increased frequency was confirmed at T1 (100%, *p* = 0.006) but not at T2. At T2, hcmv-miR-US33-5p was detected more frequently in coinfected subjects compared to monoinfected ones (100%, *p* = 0.035).

### 2.3. Inflammatory Cytokine Plasma Levels and Relationships with HCMV-Encoded miRNA Expression

**Figure 3:**
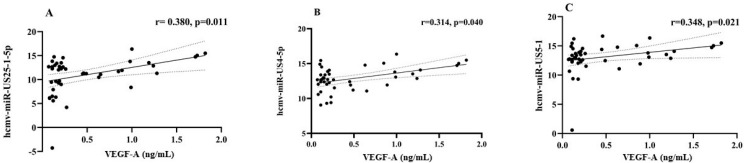
The relationship between the expression of HCMV-encoded miRNAs, which are commonly observed during lytic infection, and VEGF-A plasma levels. The correlation between the hcmv-miR-US25-1-5p expression levels and VEGF-A plasma concentrations in HCMV lytic infection (Panel **A**). The correlation between the hcmv-miR-US4-5p expression levels and VEGF-A plasma concentrations in HCMV lytic infection (Panel **B**). The correlation between hcmv-miR-US5-1 and VEGF-A plasma concentrations in HCMV lytic infection (Panel **C**). In all panels, the solid line corresponds to the linear regression line fitted to the data, while the dotted lines represent the 95% confidence interval of the regression line. Moreover, the varying hcmv-miR expression levels are presented as ΔCt, normalized to the amplification of the endogenously expressed hsa-miR-6090.

At baseline, plasma VEGF-A concentrations showed no statistically significant differences between individuals with lytic versus latent HCMV infection, with median (IQR) values of 0.21 (0.09–0.40) ng/mL and 0.19 (0.12–0.64) ng/mL, respectively. Similarly, VEGF-A levels did not significantly differ between HCMV-monoinfected individuals, median (IQR) 0.17 (0.11–0.60) ng/mL, and those HIV-coinfected, median (IQR) 0.39 (0.11–0.63) ng/mL. In the AHI group, there was no significant variation in VEGF-A levels observed between T0 and T1, with median (IQR) values of 0.17 (0.11–0.60) ng/mL and 0.22 (0.11–0.80) ng/mL, respectively. Similarly, IP-10 plasma levels did not exhibit a statistically significant difference in subjects displaying lytic versus latent HCMV infection, with median values of 0.44 (IQR 0.16–1.24) ng/mL compared to 0.26 (IQR 0.03–0.48) ng/mL. However, IP-10 plasma levels were significantly elevated in HCMV/HIV-coinfected subjects, with a median (IQR) of 0.44 (0.30–0.97) ng/mL, in comparison to HCMV-monoinfected individuals, who had a median (IQR) of 0.04 (0–0.24) ng/mL (*p* = 0.0006). These levels decreased during antiretroviral therapy at T1, with a median (IQR) of 0.11 (0.084–0.16) ng/mL (*p* = 0.0005) in comparison with T0. Among the eight HCMV-derived miRNAs more frequently detected in lytic infection, hcmv-miR-US25-1-5p, hcmv-miR-US4-5p, and hcmv-miR-US5-1 showed a statistically significant positive correlation with plasma VEGF-A concentrations (r = 0.380, *p* = 0.011; r = 0.313, *p* = 0.040; and r = 0.348, *p* = 0.021, respectively) (Figure 3A–C). However, after the false discovery rate (FDR) correction, among these three correlations, only hcmv-miR-US25-1-5p maintained its statistical significance (*p* = 0.033, as reported in Supplementary Table S1A).

In contrast, there were no significant correlations observed between these miRNA expressions and IP-10 plasma levels.

**Figure 4:**
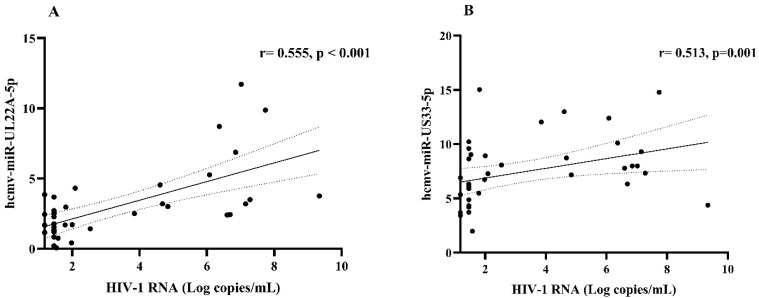
Correlation between HCMV-encoded miRNAs and HIV viremia in HIV-coinfected subjects. Correlation between hcmv-miR-UL22A-5p and HIV-1 RNA (Panel **A**). Correlation between hcmv-miR-US33-5p and HIV-1 RNA (Panel **B**). In all panels, the solid line corresponds to the linear regression line fitted to the data, while the dotted lines represent the 95% confidence interval of the regression line. Moreover, varying hcmv-miR expression levels are presented as ΔCt, normalized to amplification of endogenously expressed hsa-miR-6090.

**Figure 5:**
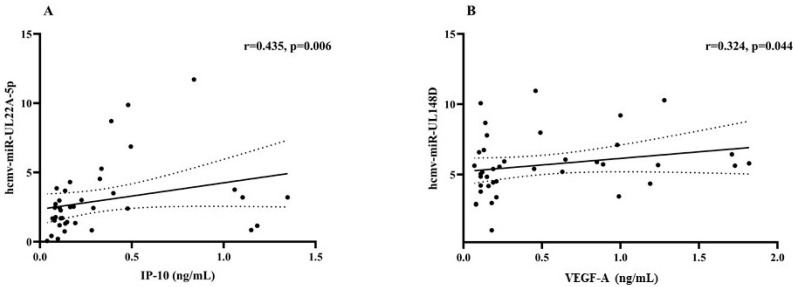
Correlation between HCMV-encoded miRNAs and inflammatory cytokine plasma levels in latently HCMV/HIV-coinfected subjects. Correlations between hcmv-miR-UL22A-5p and IP-10 plasma levels (Panel **A**). Correlations between hcmv-miR-UL148D and VEGF-A plasma levels (Panel **B**). In all panels, the solid line corresponds to the linear regression line fitted to the data, while the dotted lines represent the 95% confidence interval of the regression line. Moreover, varying hcmv-miR expression levels are presented as ΔCt, normalized to amplification of endogenously expressed hsa-miR-6090.

In latently HCMV/HIV-coinfected subjects, hcmv-miR-22A-5p and hcmv-miR-US33-5p expression levels significantly correlated with HIV-1 RNA (r = 0.555, *p* < 0.001 and r = 0.513, *p* = 0.001, respectively (Figure 4A and B)). These correlations were confirmed with FDR correction (*p* = 0.001; *p* = 0.002, respectively). Additionally, hcmv-miR-UL22A-5p expression showed significant positive correlations with IP-10 plasma (r = 0.435, *p* = 0.006, FDR-adjusted *p* value = 0.009) and hcmv-miR-UL148D with VEGF (r = 0.324. *p* = 0.044. FDR-adjusted *p* value = 0.132) (Figure 5A,B). All FDR-adjusted *p* values are reported in Supplementary Table S1B.

## 3. Discussion

The present paper demonstrates that HCMV-encoded miRNAs are differentially expressed between lytic and latent infections and that, in the context of latent infection, a different modulation in HIV coinfection is observed compared to monoinfected subjects. Taken together with our previous paper [11], which employed a completely different methodological strategy using the shot-gun Next Generation Sequencing approach of miRNA libraries comprising all viral and human miRNAs present in clinical samples, this represents, to our knowledge, the first report describing the presence of HCMV-encoded miRNA HCMV/HIV coinfection. Among the tested HCMV-encoded miRNAs, hcmv-miR-UL70-3p was consistently detected. Hcmv-miR-UL70p is part of the HCMV gene repertoire for countering cellular apoptosis and autophagy to enhance viral survival within the host [12,13,14].

During lytic infection, hcmv-miR-UL112-3p, hcmv-miR-US25-1-5p, hcmv-miR-US25-2-3p, hcmv-miR-US4-5p, hcmv-miR-US5-1, hcmv-miR-US5-2-3p, hcmv-miR-UL36-3p, and hcmv-miR-UL36-5p were statistically more frequently observed. Moreover, three of them (hcmv-miR-US25-1-5p, hcmv-miR-US4-5p, and hcmv-miR-US5-1) showed expression levels directly correlated with VEGF-A concentration in plasma. High levels of VEGF are associated with soluble biomarkers of systemic immune activation and can be generated by almost all cells under hypoxic or other stress conditions. Specifically, VEGF has profound and complex effects on endothelial cells, acting mainly as an endothelial growth factor and vascular permeating agent, contributing to vascular damage and remodeling, such as in angiogenesis [9,15]. Both ex vivo and in vitro studies indicated that hcmv-miR-US25-1 expression may accelerate atherosclerosis development and progression, likely due to the downregulation of *BRCC3*, a gene involved in DNA damage repair [16]. Hcmv-miR-US4-5p and hcmv-miR-US5-1 contribute to processes such as immune response evasion, the inhibition of autophagy and apoptosis, and a reduction in inflammatory cytokine production, depending upon the cell type investigated and cell activation state [17].

The differential HCMV-miRNA expression during latency between monoinfected and HIV-coinfected subjects and its association with inflammatory activation is notable. During HCMV latent infection, HIV-coinfected subjects had higher detection frequencies of hcmv-miR-UL22A-5p, hcmv-miR-UL148D, and hcmv-miR-US33-5p. Hcmv-miR-UL22A-5p was directly correlated with the plasma concentration of IP-10, while hcmv-miR-UL148D expression was directly related to VEGF-A plasma levels, at least without the multiple comparison correction. It is noteworthy that hcmv-miR-UL22A-5p and hcmv-miR-US33-5p were also associated with HIV-1 viremia and therefore could be controlled by antiretroviral therapy, whereas hcmv-miR-UL148D, associated with VEGF-A expression, seems to be less linked to HIV viremia.

In solid organ transplanted individuals, hcmv-miR-UL22A independently predicted the recurrence of HCMV viremia upon the discontinuation of antiviral therapy and could be evaluated further as a biomarker. Hcmv-miR-US33-5p expression facilitates the establishment or maintenance of HCMV latency [18]. Hcmv-miR-US33-5p was found to influence the apoptosis of human aortic vascular smooth muscle cells and was more abundant in the plasma of patients with acute aortic dissection [19]. Hcmv-miR-UL148D is one of the key HCMV-miRNAs involved in establishing and maintaining viral latency [20]. It accumulates abundantly during the late stages of HCMV infection, and its mechanism for promoting latency was explained in [21]. Additionally, hcmv-miR-UL148D inhibits staurosporine-induced apoptosis [22,23]. In the context of immune modulation, it has been reported that miR-UL148D mediates RANTES downregulation in human foreskin fibroblast (HFF) cells after infection with the Toledo strain but not the AD169 strain [24]. Moreover, in primary myeloid cells, hcmv-miR-UL148D inhibits the activin-A triggered secretion of IL-6 [25]. Further research is needed to elucidate hcmv-miR-UL148D’s wide spectrum of targets and the pathways involved in its expression, which could clarify the discordant results across different studies

## 4. Materials and Methods

### 4.1. Study Population and Virological Evaluation

**Figure 6:**
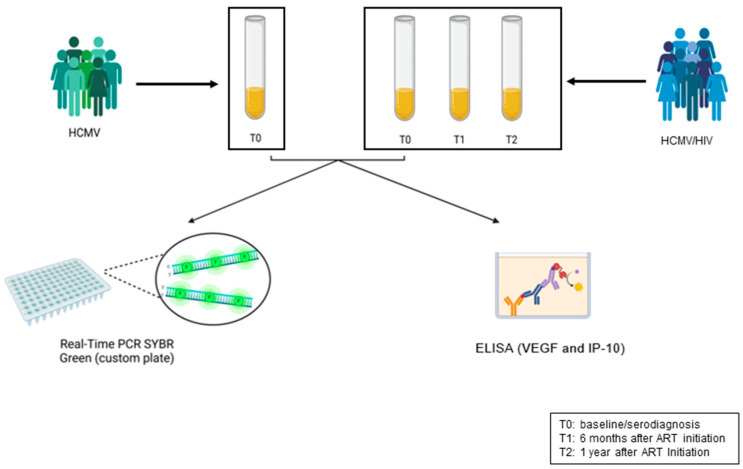
Diagram describing analytical workflow. Designed with Biorender (https://www.biorender.com/, last accessed on 6 June 2025).

Sixteen individuals with AHI from the National Institute for Infectious Diseases (INMI) observational cohort of primary infection (SIREA cohort), who tested positive for HCMV (IgG anti-HCMV-positive), were evaluated at the time of HIV diagnosis (T0) and after 6 (T1) and 12 months (T2) of early antiretroviral therapy (initiated within seven days from serodiagnosis) (Figure 6). At these times, both HIV and HCMV viremia were evaluated. As a control group, 14 subjects who tested positive only for HCMV serology were included. HCMV IgG levels were quantified with LIAISON^®^ kits on LIAISON^®^ XL (DiaSorin, Saluggia, VC, Italy). The serodiagnosis of AHI subjects was posed based on either the combination of an HIV Ab/Ag Combo positive (Abbott ALINITY HIV Ag/Ab Combo assay, Abbott Park, IL, USA) and an Immuno Blot (Geenius, BioRad, Bio-Rad Laboratories S.r.l., Milan, Italy) negative assay with positive HIV-1 RNA or an HIV Ab/Ag Combo positive and Immuno Blot indeterminate assay, according to WHO criteria for Western blot confirmatory assay, i.e., two Env-reactive bands. Plasma HIV-1 RNA was measured by Aptima™ HIV-1 Quant Dx Assay (Hologic, Inc., San Diego, CA, USA). All subjects were also tested for HCV Ab and HBsAg/anti-HBV core antigen determinations using ALINITY I HCVAb, HBsAg qualitative, and HBc tot RGT, respectively (Abbott Diagnostics, Chicago, IL, USA). HCMV DNA evaluation was performed on whole blood by HCMV ELITE MGB on the ELITe InGenius Instrument (ELITech Group S.p.A, Turin, Italy). The SIREA study was approved by the INMI Ethical Committee on 18 February 2014, while control samples were residuals of diagnostic evaluations (Ethical Committee, CET Lazio area 4 approval number 61/2023, 4 December 2023). All subjects were requested to sign an informed consent form.

### 4.2. MiRNA Detection

To purify cell-free total RNA, including miRNA and other small RNA, from 200 µL of plasma, the miRNeasy Serum/Plasma Advanced Kit (QIAGEN, Hilden, Germany) was utilized. Reverse transcription was performed using the miRCURY LNA RT Kit (QIAGEN), adding the small RNA UniSp6 spike-in as a control of retro-transcription. Quantitative, Real-Time PCR was performed using miRCURY LNA miRNA Custom PCR Panel (QIAGEN) and miRCURY LNA SYBR Green PCR Kit (QIAGEN) on the Applied Biosystem ABI 7900HT Fast Real Time PCR System (Thermo Fisher Scientific Inc., Waltham, MA, USA). A custom PCR panel was used, specifically designed to contain a single specific miRCURY miRNA PCR primer (QIAGEN) in each well to amplify the following fourteen HCMV-encoded miRNAs: hcmv-miR-UL112-3p, hcmv-miR-UL112-5p, hcmv-miR-UL148D, hcmv-miR-US25-1-5p, hcmv-miR-US25-2-3p, hcmv-miR-US33-5p, hcmv-miR-US4-5p, hcmv-miR-US5-1, hcmv-miR-US5-2-3p, hcmv-miR-UL22A-3p, hcmv-miR-UL22A-5p, hcmv-miR-UL36-3p, hcmv-miR-UL36-5p, and hcmv-miR-UL70-3p. The panel also used primers to amplify UniSp6 RNA and the human miRNA hsa-miR-6090, chosen for its stable expression in various plasma samples as a reference for normalizing HCMV miRNA quantification [26]. Samples displaying less than 42 cycles were considered positive for the specific miRNA target of the amplification. This threshold was set on the basis of a number of replicates of negative controls to ensure false positive detections less than 5%. Hsa-miRNA-6090 and UniSp6, along with primer and amplification reagents, were used as representative assays for miRNA threshold detection. All miRNA Ct values are displayed in Supplementary Table S2A–C. MiRNA expression levels were calculated using the delta-cycle threshold (ΔCt) method using Ct values for both UniSp6 (calculated as ΔCt = Ct target hcmv-miRNA–Ct UniSP6) and the endogenous human miRNA miR-6090 (hsa-miR-6090) (calculated as ΔCt = Ct target hcmv-miRNA–Ct hsa-miR6090). Only significant correlations between miRNA expression levels and cytokine amounts or HIV-1 RNA copies/mL detected using both UniSp6 and hsa-miR-6090 were considered and presented.

### 4.3. Cytokine Quantification

The plasma levels of VEGF-A and IP-10 were quantified using ELISA immunoassays: human VEGF-A ELISA Kit (antibodies.com, Cambridge, UK) and Human IP-10 ELISA Kit (antibodies.com (accessed on)). VEGF-A and IP-10 concentrations in plasma were calculated according to the manufacturer’s protocol instructions.

### 4.4. Statistical Evaluation

Statistical evaluation was performed using both descriptive and inferential methodologies. Comparative analyses were executed using Fisher’s exact test to highlight differential miRNA detection frequencies between HCMV lytic and latent infection phases, as well as between HCMV-monoinfected and HCMV/HIV-coinfected subjects. A Mann–Whitney comparative test was performed to evaluate differences in plasma cytokine levels among groups of subjects, while the Wilcoxon matched-pairs signed-rank test was conducted to evaluate differences in both median HIV viremia and plasma cytokine levels over the follow-up period. Spearman’s correlation analyses were performed to evaluate the relationship between cytokine plasma concentrations and HIV plasma viral load and the relationships between cytokine plasma levels and HCMV-encoded miRNA expression. To account for multiple comparisons, the *p* values obtained from the correlation analysis were adjusted using the Benjamini–Hochberg FDR correction. FDR-adjusted *p* values < 0.05 were considered statistically significant. All statistical procedures were carried out using GraphPad Prism v.10.2.1 (GraphPad Software. Boston, MA, USA). Statistically significant *p* values were 0.05 and 0.01.

## 5. Conclusions

HCMV-encoded miRNAs are modulated differently during HCMV lytic and latent infection. In HIV/HCMV-coinfected subjects, hcmv-miR-UL22A-5p, hcmv-miR-UL148D, and hcmv-miR-US33-5p were upregulated in the absence of HCMV replication. While hcmv-miR-UL22A-5p and hcmv-miR-US33-5p were strictly correlated with HIV-1 RNA and their expression may be controlled by ART, hcmv-miR-UL148D, correlated to the production of the inflammatory marker VEGF, seems to be less linked to HIV-1 plasma levels, and it could be overexpressed even during antiretroviral therapy.

A deeper understanding of HCMV-encoded miRNA biology may facilitate the comprehension of the HCMV/HIV coinfection pathogenetic mechanisms.

## Figures and Tables

**Table 1:** The demographics of the study population. The demographics of HCMV/HIV individuals are reported in Panel A. * For both HCMV DNA and HIV-1 RNA, not detected values are indicated as ND. The Log of half the value of the limit of detection (LOD) of the used assays was assigned to perform statistic evaluation, ** whereas for detected values below the limit of quantification, a number corresponding to one unit below the limit of quantification was assigned. T0 is the time of serodiagnosis. T1 is 6 months and T2 is 12 months after antiretroviral therapy start. Antiretroviral treatment was initiated in all subjects within one week from serodiagnosis. NA means not available. The demographics of HCMV-monoinfected individuals are reported in Panel B. * For HCMV DNA, not detected values are reported as ND. The Log of half the value of the LOD of the used assays was assigned to perform statistic evaluation.

**A**							
**Sample**	**Age**	**Sex**	**CD4 T cells/mm^3^**	**HCMV DNA (Log IU/mL)**	**HIV-1 RNA** **T0 (Log cp/mL)**	**HIV-1 RNA** **T1 (Log cp/mL)**	**HIV-1 RNA T2 (Log cp/mL)**
AHI 1	30	M	546	ND *	6.60	2.09	NA
AHI 2	49	M	540	R < 2.25 **	6.00	R < 1.46 **	ND
AHI 3	30	M	785	ND	3.85	R < 1.46	ND
AHI 4	36	M	414	ND	6.37	R < 1.46	NA
AHI 5	42	M	274	ND	7.15	2.16	NA
AHI 6	50	M	194	ND	7.74	1.58	R < 1.46
AHI 7	29	M	413	ND	4.61	R < 1.46	NA
AHI 8	39	M	425	ND	7.02	R < 1.46	R < 1.46
AHI 9	33	M	669	ND	4.68	R < 1.46	ND
AHI 10	60	M	138	ND	6.08	2.00	R < 1.46
AHI 11	46	M	260	ND	6.85	1.81	R < 1.46
AHI 12	49	M	192	ND	6.69	1.98	2.54
AHI 13	33	M	524	ND	7.28	1.53	R < 1.46
AHI 14	40	M	672	ND	4.84	R < 1.46	R < 1.46
AHI 15	55	M	349	ND	9.34	ND	ND
AHI 16	53	M	509	5.51	6.70	1.79	R < 1.46
Median (IQR)	41 (33–50)	16 M	420 (264–545)	1.73 (1.73–1.73)	6.65 (5.13–7.12)	1.50 (1.46–1.94)	1.46 (1.18–1.46)
**B**							
**CTR**		**Age**		**Sex**		**HCMV DNA (Log IU/mL)**	
CTR 1		47		F		ND *	
CTR 2		45		F		ND	
CTR 3		57		F		ND	
CTR 4		32		F		ND	
CTR 5		41		M		ND	
CTR 6		44		F		ND	
CTR 7		34		F		ND	
CTR 8		37		F		ND	
CTR 9		54		F		ND	
CTR 10		39		F		ND	
CTR 11		64		M		3.30	
CTR 12		63		F		3.67	
CTR 13		48		M		5.59	
CTR 14		64		F		3.86	
Median (IQR)		46 (39–59)		11 F/3 M		1.73 (1.73–3.39)	

## Data Availability

Data is contained within the article and Supplemental Materials.

## Supplementary Materials

The following supporting information can be downloaded at https://www.mdpi.com/article/10.3390/ijms26125673/s1.
